# Challenges of performing essential public health functions by the physicians at leadership positions in peripheral level government health system in Bangladesh: A qualitative exploratory study

**DOI:** 10.1371/journal.pone.0268026

**Published:** 2022-05-12

**Authors:** Dipika Shankar Bhattacharyya, Sohana Shafique, Iffat Nowrin, Iqbal Anwar

**Affiliations:** Health Systems and Population Studies Division, icddr,b, Dhaka, Bangladesh; University of Calcutta, INDIA

## Abstract

**Introduction:**

Identifying and ensuring the Essential Public Health Functions (EPHFs) is one of the core agendas of the World Health Organization to strengthen the health system of a country. The definition of EPHFs varies widely, considering country needs. In Bangladesh, the physician cadres are in the leadership position to oversee the EPHFs at the district and sub-district levels. However, there is a dearth of contextual information regarding the purview of essential EPHFs in the country. The purpose of this study was to document the perception of key stakeholders on what constitute the EPHFs at district and sub-district level and identify the challenges they face in providing the services.

**Methods:**

We carried out a qualitative exploratory study consisting of document review and Key Informant Interviews (KIIs). The desk review included the organograms of the government district and sub-district level health facilities and the job description of Civil Surgeons (CSs) and Upazila Health and Family Planning Officers (UHFPOs). In addition, 15 KIIs with relevant professionals and stakeholders from the Directorate of General of Health Services (DGHS), Ministry of Health and Family Welfare (MoHFW) were conducted. Collected data were analyzed thematically.

**Results:**

Three major categories of EPHFs were identified: i) population-oriented preventive functions, ii) clinical preventive functions, and iii) administrative/management functions. The CSs and UHFPOs need to ensure these wide range of EPHFs at the district level and below. However, at peripheral level, the leadership positions’ clinical and public health roles often get amalgamated. Therefore, ensuring public health functions are hampered. Besides, these positions need training and adequate support staff to perform the EPHFs effectively.

**Conclusion:**

Recognizing the EPHFs in the Bangladesh context is crucial. Revisiting the job descriptions and strengthening appropriate public health services at different tiers in the country health system should be prioritized to achieve health-related Sustainable Development Goals.

## Introduction

The conceptual features of Public Health are becoming increasingly complex across the globe [1–3]. During the late 19^th^ and early 20^th^ century, the idea of core public health functions was concentrated on ensuring hygienic practices through maintaining environmental sanitation to control communicable diseases [4,5]. However, over time, the academic and practical purview of public health has expanded enormously and now encompasses addressing the epidemiological transition to prevent and control non-communicable diseases (NCDs), which require coordinating with a wide range of ’non-health’ sectors both globally and locally [6]. The broader spectrum of the "public health" concept is thus devoid of any precise boundary for its practice [7]. Especially, as a professional discipline, public health lacks clear definition and support within the governmental service delivery structure in many low- and middle-income countries [8].

In response to this growing complexity regarding the concepts of public health, academicians and global health leaders felt the need to develop a list of essential public health functions that are indispensable to protect and promote population health [9]. The Centers for Disease Control and Prevention (CDC), USA, developed a list of essential public health functions in 1994 [10]. Subsequently, the World Health Organization (WHO) developed a similar list in 1997, mainly in response to the needs of the newly independent countries by the dissolution of the former Soviet Union [9,11]. Since then, over the last 25 years, the WHO regions and national governments have developed their list of region/country specific Essential Public Health Functions (EPHFs). While these lists share some commonalities, there are considerable differences as well, since the EPHFs are actually embedded within the country’s broader health system contexts and health needs of their citizens [11].

In Bangladesh, the Ministry of Health and Family Welfare (MOHFW) is responsible for providing public health services, including health promotion, disease prevention and curative care [12]. The MOHFW consists of several directorates, institutes, units, and cells; all of which have specific public health functions e.g., service delivery, disease control, drug supply management, research and training, regulatory tasks, health financing issues, and others. However, in Bangladesh, the health care service delivery is mainly maintained through two directorates of MOHFW. The Directorate General of Family Planning (DGFP) is ensuring family planning services across the country, and the Directorate General of Health Services (DGHS) holds the responsibility to provide health care services through a country-wide tired network of health centers and hospitals [13]. It is worth noting that Bangladesh inherited a centralized health system from the British colonial period [12]. The Bhore Committee Recommendations in 1946 to integrate curative and public health services [14] are still followed in the country. As a result, the public health services are amalgamated with the curative health care service particularly at operational levels.

Since the curative and public health services are not differentiated, there is a lack of clear understanding and documentation on what constitutes the EPHFs in the country context. According to the current organizational structure of the DGHS, the government physician cadres are at the leadership positions at the sub-district and the district level to coordinate all public health services [12]. At the district level, the Civil Surgeons (CSs), and at the sub-district level, the Upazila Health and Family Planning officers (UHFPOs) are responsible for overseeing and managing preventive and curative services. The previous study indicated that generally, these physician cadres lack appropriate knowledge or expertise in the area of Public Health or ’Management’ [15]. However, there is a dearth of context-specific literature on what constitutes EPHFs and what challenges physician leaders face in carrying out them on the ground. From this consideration, the aim of this paper is twofold: 1) to create solid evidence-base about the perception of key stakeholders regarding what constitutes the EPHFs, and 2) what challenges do the physician leaders face in performing these EPHFs at the district and sub-district level of the national health systems.

### Conceptual framework

**Fig 1:**
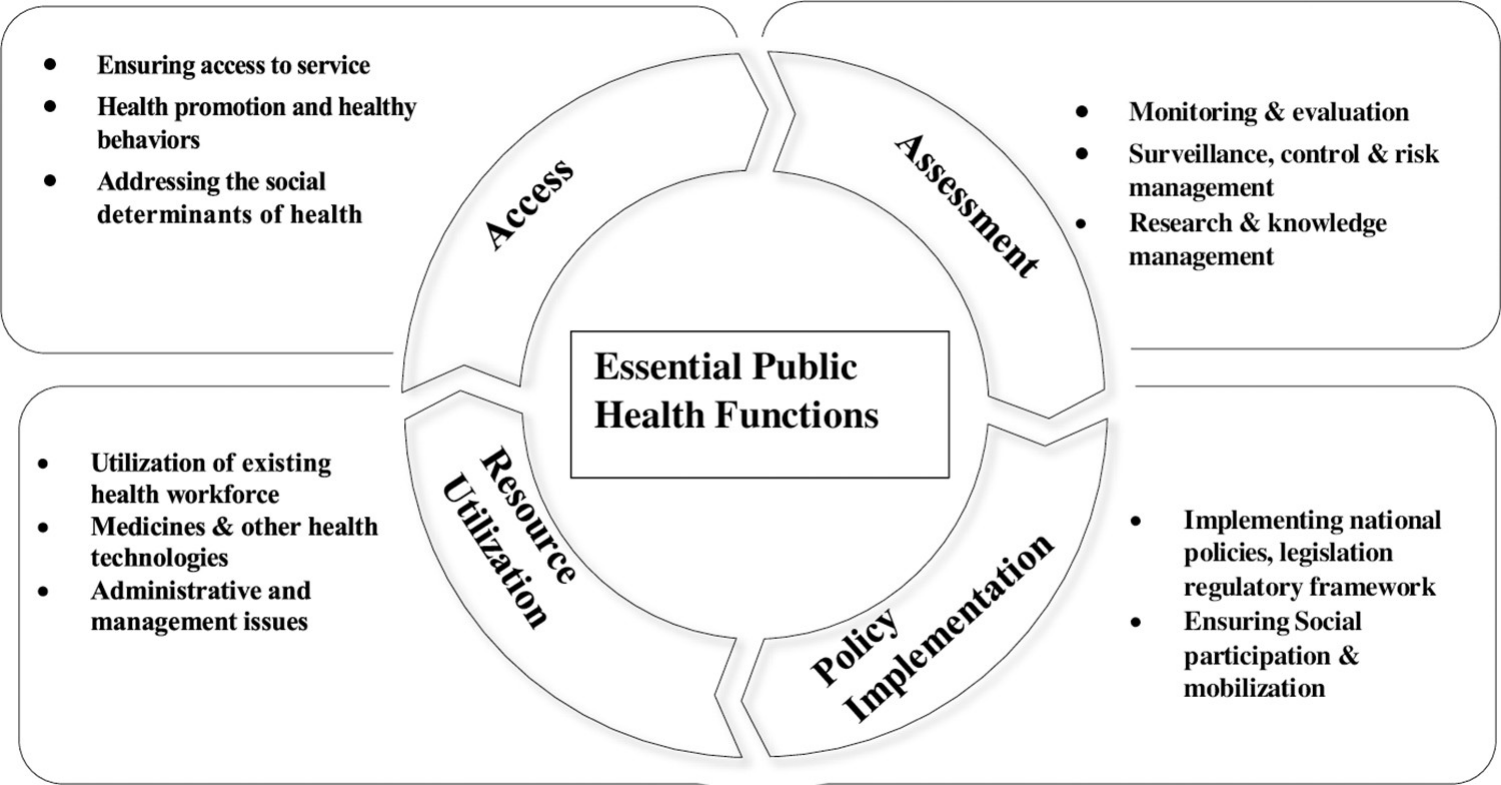
Conceptual framework for the essential public health functions.

To relate with current scholarship and explanation of a qualitative research, a conceptual framework is built upon existing theory, literature and experience of researchers [16]. From this perspective, we developed a conceptual framework (Fig 1) to address the study objectives. The conceptual framework is mainly adapted from ‘The Renewed Framework for the Essential Public Health Functions’ published by the Pan American Health Organization (PAHO) [17]. Adopting a right-to-health approach, the PAHO framework incorporated four steps- a) assessment; b) policy development; c) resource allocation; and d) access. The ‘assessment’ step includes analysis of the population health problems and corresponding them with the country health systems limitations. The next step, the ‘policy development’ deals with course of action to address the challenges by ensuring dialogues and participation of relevant stakeholders. The ‘resource allocation’ refers to disbursement of critical resources required. Finally, the access stage, ensures conditions of equitable and universal access to health [17,18].

The PAHO framework, however, is primarily applicable for national governments to strengthen their essential public health functions at the population level. Since our study focuses on a specific tier of health system and on particular type of health cadre in Bangladesh, we adapted the framework to fit Bangladesh district health systems context. For example, considering the fact that the physicians at the leadership positions at districts and sub-districts in the country are not allocating resources [15], we modified this as “Resource utilization”. Accordingly, we incorporated how they are receiving and utilizing the allotted resources, and addressing the administrative and financial issues as part of EPHF. Besides, we proposed “policy implementation” instead of “Policy development” for the same reason.

## Methods

### Study design, settings, sampling strategy

This exploratory study was conducted in Bangladesh between October 2018 and September 2020 where documentary review and qualitative in-depth interviews were the key data collection methods. We chose an exploratory design as it enables researchers to look into complex patterns of perceptions and experiences of the relevant stakeholders and thus help develop a deeper understanding of the contextual and health system factors [19].

This study was conducted mainly in Dhaka, the capital of Bangladesh since the MOHFW and DGHS is located here where majority key study participants work, and access to them was easy. Furthermore, Narayanganj, Rangamati, and Dinajpur districts were selected for sub-national data collection to capture intra-country variations in public health functions and their implementation challenges.

For the desk review, the document search was conducted on the websites of MOHFW and DGHS to collect the organogram of district and sub-district level health facilities and job descriptions of relevant staff. In addition, a snowballing technique was applied for searching some policy documents in the library and other repositories which were not available online. This study reviewed the job descriptions of CS, UHFPO, and organogram of district and sub-district (known as Upazila) level health-facilities [20–23].

A total of 15 Key Informant Interviews were conducted with participants from MOHFW, DGHS, and district and sub-district health offices. In addition to the government stakeholders, we also interviewed representatives from academicians, development partners, and other key stakeholders such as individual consultants. The list has been provided in Table 1. The sample was drawn employing the purposive sampling strategy as well as the snowball technique. Initially, a list of key informants was developed based on meetings with the study team members and experts who have extensive experience in working with Human Resources for Health issues in Bangladesh. This list was further expanded as per the suggestion of the key informants since after each interview, they were asked to suggest potential informants who might be resourceful for the study. To determine the sample size, the idea of "information power" was utilized in this study. Malterud et al. (2016) suggested that the more depth of information the study participants hold in line with the research question, the smaller sample size is needed [24].

**Table 1:** Sample size and type of key informants participated.

Type of Key Informants	Sample size (n)
High officials from MOHFW and DGHS	3
Civil Surgeons, UHFPOs, Medical Officers	6
Academicians, Researchers	3
Development partners	3
**Total**	**15**

### Data collection

Trained anthropologists conducted KIIs under the guidance of two supervisors with extensive knowledge on Bangladesh health systems, and technical skills for qualitative research. The data collectors used semi-structured interview-guideline to facilitate KIIs, which was developed and finalized by rigorous review by the study investigators. The respondents were asked to identify the essential public health functions at the district and sub-district levels. Besides, the existing challenges that the physician cadres at the leadership position face in carrying out the essential public health functions, were also explored. The interviews were conducted in Bangla and lasted from 45 minutes to 1 hour, and were recorded digitally upon receiving the interviewee’s written consent. In each of the interviews, two researchers were involved in facilitating and note-taking. If the key informant did not provide consent for recording, extensive note-taking was organized. The recorded interviews were verbatim transcribed immediately and then translated into English for further analysis.

### Data analysis

We used the standards for reporting qualitative research (SRQR) checklist [25] to organize and report the qualitative data collected in this study. To address study objectives, a thematic analysis was undertaken employing codes and data displays. Prior to data collection, a codebook was prepared with the definition of a-priori codes, based on the semi-structured guideline. As the study progressed, the codebook was modified and expanded based on the emergent codes. All transcripts were compiled and repeatedly read for data familiarization. A team approach was undertaken to minimize bias, the two researchers coded the same transcript separately, and then any discrepancy was minimized through meetings among of study investigators. The transcripts were coded in Atlas.ti version 5.2, and emerging codes from the transcripts were defined and applied as the analysis proceeded. All the apriori and emerging codes were then arranged in data matrices to identify patterns, commonalities and differences.

### Ethical considerations

The protocol of this study was approved by the Institutional Review Board of International Center for Diarrheal Disease Research, Bangladesh (known as icddr,b). The IRB approved protocol number is PR-19008. Before each interview, informed consent and permission for audio-recording were obtained in writing from the Key informants. Data confidentiality is maintained. The data collectors described all elements of consent to study respondents orally to clarify the purpose of the research. For ensuring anonymity and confidentiality, all transcripts were de-identified with a combination of numbers and letters. All data were kept in locked storage, or controlled access folders, allowing only investigators of the study and members of the ERC of icddr,b to access information, if needed.

## Results

The results are organized into two broad themes. The first theme describes what constitutes the essential public health functions at the district and sub-district level of Bangladesh, as perceived by the key stakeholders. The second theme illuminates the challenges regarding performing those functions.

### Essential public health functions

The desk review reveals that the Civil Surgeon and the UHFPO are mainly assigned to ensure coordination of all tasks under the MOHFW within their jurisdiction. The 13-point job description for CS includes supervising all activities of the government and non-government health facilities, supporting UHFPOs in implementing public health programs and settling the medico-legal issues. The CS is also responsible for implementing all the health-related policy decisions at the district level with authority to oversee all administrative and financial matters of MOHFW staff. Besides, the CS is mandated to synchronize the tasks to ensure safe water and waste management with other government bodies, maintain district reserve store for health, and prepare and respond to epidemics and other health emergencies. Similarly, the UHFPO is responsible for coordinating all health and family planning activities at the sub-district level.

From the KII, we wanted to explore how the key stakeholders define the EPHFs; one UHFPO mentioned,

*"Public health functions are those focused on prevention; that is, before any disease happens, we need to predict this and take preventive measures. So except for the hospital-based clinical services, all the services are public health works. For example, public health works are telling mothers to take four antenatal care (ANC) services and raise awareness about five danger signs. Besides, establishing referral linkage is also a public health work. Vaccination and activities related to Extended Program on Immunization (EPI) are public health functions." (KI, UHFPO)*

All of the key informants reflect this view on the prevention of disease as the key public health function. According to the key informants, the prevention activities broadly include ensuring immunization, health education, managing government campaigns (de-worming campaign) at the district and sub-district level. The prevention is also multifaceted because different type of diseases necessitates different sets of interventions for prevention. Considering the existing disease burden of Bangladesh, one KI categorized the prevention activities in different sets, as quoted-

*"If you see at the Upazila and below level, the public health functions will be the water and sanitation, immunization, antenatal and postnatal care issues, Tuberculosis (TB) screening. Now Non-Communicable Diseases (NCD) are rising, so NCD screening and referral are also public health issues in Bangladesh. Besides, sexual and reproductive health, especially family planning issues, are major public health functions. So family planning, sexual and maternal health, antenatal care these constitute one basket while NCD is another basket and child immunization and child health and nutrition is another basket." (KI, Development Partner)*

To assure these prevention measures are appropriately taken, the public health leaders need to ensure the execution of national health programs, monitor the disease pattern, and get regular data update from the field level. As a result, the physician public health leaders have identified maintaining close coordination and supervising every facility within the jurisdiction area as an essential public health function. For example, one UHFPO mentioned-

*"As a UHFPO, I have to manage 17 union sub-centers and I have to look after the EPI activities in my area; I also need to supervise and monitor all the community clinics; these are public health work." (KI, UHFPO)*

The administrative aspect of public health functions has been emphasized by the district-level managers also. For example, one of the CS said:

*"As Civil Surgeon, we provide health education to people… In addition, I manage a financial unit and need to work as an officer responsible for the development and salary of certain staff. Furthermore, I manage a central store through which I maintain all the Upazila (sub-district) in procuring drugs and other logistics. I also ensure the accountability of all the existing services-preventive, promotive and curative- I ensure that these services are being properly ensured at all levels in my district. Besides, I also need to raise awareness and check whether any new disease emerged in my district. These works are public health works." (KI, CS)*

The KII finding also suggests that at the divisional and national level, ensuring proper administrative management and providing regular feedback to the policymakers for formulation or update of health policy are the main public health functions. Therefore, both from the desk review and KIIs we summarized the public health functions as provided in Table 2.

**Table 2:** List of essential public health functions as perceived by key informants.

Categories of essential public health functions performed by physicians		
Population-oriented preventive functions	Clinical-based preventive functions	Administrative/management functions
Monitoring health conditions of population	Implementing EPI program	Supervision of health facilities, regular monitoring of field activities
Outbreak control activities	MNCH services	Financial management
Social Behavior Change Communication (SBCC) for Maternal Neonatal and Child Health (MNCH), Water Sanitation Hygiene, Nutrition, Family Planning (FP) etc.	De-worming, Vitamin A Plus campaigns, etc.	Vertical programs, observing weeks /days
Nutrition Services	Screening for Tuberculosis (TB) and Non Communicable Diseases (NCD), FP	Central store management; Logistics and transport management;
Community engagement/School-health	Hospital administration, waste management	Supporting Policy level decisions; Managing operational Plan/Sector Program to achieve Sustainable Development Goals (SDGs)

### Challenges of performing essential public health functions

The majority of key informants (n = 12) identified that public health works require a different set of expertise than clinical works. The analytic mindset and having a grasp of statistical analysis is necessary for appropriately completing this public health leader job. One KI mentioned:

*"It (Public Health) is like television. The display or the monitor in the front does not show the whole thing—vast machinery is working in the background. Similarly, seeing patients and giving prescriptions to the patients is not the whole thing, the majority is the public health works behind this. …I would say there is a need for persons with understanding on epidemiology, infectious disease control, research, health policy, and systems issues." (KI, Researcher)*

However, according to the key informants, the physicians need to go through a long career to get promoted in public health leadership positions at the district and sub-district level (e.g., CS / UHFPO). During the long years of their early career, they serve as clinical caregivers; little scope to apply their public health knowledge obtained during graduation. As a result, their knowledge and skills in the field of public health and epidemiology is not up-to-date and are dependent on other resources for essential (or emergency) public health functions; one CS in this regard mentioned:

*"At our medical school, we learned about the definition of rate, ratio, endemic, pandemic, etc. But we forget these while performing our everyday activities. For example, if there is an emerging disease like dengue, as a CS, I manage it as per the instruction provided by the Institute of Epidemiology, Disease Control and Research (IEDCR). They send specialists, and then they tell us what to do and how to do it. We need to learn from them first and work accordingly. If we had trained people here, then we could have handled beforehand and could manage this efficiently." (KI, Civil Surgeon)*

The lack of adequate training in public health and epidemiology is one of the most highlighted challenges found in Bangladesh’s health systems context. As one UHFPO mentioned-

*"As a medical officer, before getting the 6th grade, one is mainly involved in seeing the patient at the hospital outdoor or emergency department..; it would be better if at least they are provided with training for one or two months before they assume to this position." (KI, UHFPO)*

While this reflects the demand for particular skills in performing public health functions, it also focuses on the fact that the physician cadres predominantly focus on the clinical side of the job at the district and sub-district level before assuming the post responsible for leading the public health activities. The amalgamation of clinical and public health services is therefore emphasized as a challenging issue, that is more evident as one UHFPO mentioned-

*"As a medical officer, I see patient. But back in my mind, I am restless; I appear for postgraduate admission, one after another. Say, tomorrow I will sit for pediatrics, the day after tomorrow for medicine, and the next day for surgery. When nothing happens, I become frustrated. Only then do I go to my authority and say, ’Sir, I want to be a UHFPO now." (KI, UHFPO)*

The challenges were also found regarding the shortage of human resources for performing the public health functions at the district and sub-district levels. According to the current practice of work distribution at the sub-district, the UHFPO assigns one medical officer to facilitate the regular coordination of vertical health programs like TB control, Malaria control, EPI, etc. This medical officer is designated as Medical Officer Disease Control (MODC), responsible for a regular field visits to the community clinics and ensuring data flow. However, our desk review finding suggests, in the current government-approved organogram of Upazila Health Complex, there is no post sanctioned for MODC. The absence of a sanctioned MODC is mentioned as a challenge by several key informants (n = 4); for example, one UHFPO said:

*"A post for MODC is there at Upazila level, but this is not a sanctioned one. Among the Medical Officers, one is assigned to perform the functions of MODC. But how could he do these jobs? This person has a regular duty outdoor. So if he goes outside to visit the community clinic or EPI activities in the field; who will run the show at outdoors? For these reasons, you cannot make him accountable." (KI, UHFPO)*

The inadequate human resource for performing public health functions is also found at the district level, highlighted by another key informant from MOHFW as-

*"At the district level, the Civil surgeon does not have enough human resources to conduct the public health activities. In addition, deputy civil surgeon is not there at every place, and the statistician is not oriented with epidemiology to understand context-based disease pattern." (KI, high official)*

This quote represents that there is also a challenge with the existing inappropriate skill mix. The public health functions necessitate data-driven decision where the role of a trained statistician with proper understanding of epidemiology is important, which is missing in many cases.

## Discussion

The study is one of its first kind in reporting what constitutes the public health functions in Bangladesh and what challenges the physician cadre in the leadership positions at district and sub-district levels face in carrying out public health functions in the context of country health systems. The finding suggests that ensuring preventive services and coordinating with a broad range of stakeholders across and beyond the health system are the primary public health functions at the district and below. This study is focused on district health systems in Bangladesh. The available evidence on public health functions mostly covers country-wide rather than any specific tier of the health system. Nevertheless, the study finding is somewhat similar to evidence from other contexts in the globe. For example, our essential function results resemble the list of 10 public health functions delineated by the CDC-USA [10]. The results are also align with the 11 public health functions of the PAHO framework which was adapted for our conceptual framework. However, the CDC list contains component on developing the public health workforce, which was not found in our study as we focused on district and below. The World Health Organization (WHO) outlined eight essential public health functions for the countries of the Eastern Mediterranean region. Among these eight functions, four were regarded as core functions that comprised of disease surveillance, emergency preparedness, health protection and safety, and health promotion; while the other four were considered as enabling functions of public health, which consisted of ensuring stewardship, maintaining quality health-workforce, social mobilization and public health research [26]. Except for the workforce and research issues, we have found similar results in emphasizing preventive services and maintaining coordination. According to the conceptual framework we developed, research and knowledge management was one of the key components of essential public health function. However, the key informants in this study did not mention research as public health function, probably because there is a general lack of research culture in the government health system, particularly at the district level and below [27]. Although the situation is currently improving and increasing research-policy communication is occurring, still, contributions in generating research evidence from public sector institutions are inadequate in Bangladesh [28].

Regarding the challenges, the study result showed that skill-retention around public health is a significant challenge for performing public health functions by the physician leaders. The finding suggests that due to the amalgamation of clinical and public health cadres at the sub-district and district level, a physician needs to spend a substantial amount of their career doing clinical works before assuming the post of public health leader. As a result, they often drop the degree of academic competence required to perform public health functions. This finding corroborates with the findings from Joarder et al. (2018) that suggested that career for medical doctors in the Bangladesh health system is an assortment of clinical, education, and public health track; and there is a provision of lateral entry to public health track from other tracks [29]. The previous study also confirmed that the progression for physicians who pursue a career in the public health track in the peripheral tier of health system, is comparatively slower than the other tracks [30]. Besides, our result is similar to previous research that indicated a general paucity of public health expertise among Bangladesh’s district and sub-district levels [15].

Regarding human resources, our study finding suggested that the challenges are around the lack of sanctioned public health positions at the district and sub-district level for performing public health functions. According to the conceptual framework, utilization of existing health workforce for performing essential public health function was highlighted. The study results also showed that in the absence of required sanctioned human resources, the physicians at public health leadership positions were adopting various strategies. Previous studies and government documents showed a general shortage of doctors, inappropriate skill mix, and inequitable distribution in the health system of Bangladesh [12,31–33]. However, limited evidence in the Bangladesh context explicitly focuses on the shortage of physician cadres for essential public health functions. Joarder et al [29] suggested that there is limited scope to draw public health experts for specific public health positions in the health system in the current government recruitment mechanism. This is well-reflected in our study finding as well.

Our study finding also suggested a need for capacity building for the district and sub-district level health managers on public health issues once they assume these posts. The need for continuous training is highlighted in many previous studies from other countries as training facilitates the public health leaders to improve their skills and knowledge for adapting to the evolving nature of the public health landscape [34]. Public health professionals with proven public health knowledge and expertise are crucial to improving health system performance in line with UHC and global health security [35]. Findings from similar country settings also showed that the skilled public health physician cadres at the lower tier of the health system could be the change-maker with improved analytical competencies, leadership, context-specific planning, and management [36–38]. Therefore, the results of our study indicate that there is a need for policy attention on addressing the existing challenges that the public health leaders are currently facing in the district and sub-district levels in Bangladesh.

The findings of our study need to be considered in the context of some limitations. Our sample size was appropriate enough in line with the research question to be answered, but it may not be interpreted with generalizability for the total health system of Bangladesh. Especially, this study is focused only on the public health leadership positions, at district and below, working under the umbrella of the Directorate General of Health Services (DGHS), MOHFW. The other directorates, such as Directorate General of Family Planning (DGFP), Directorate General of Drug Administration (DGDA), or other Ministries that perform certain EPHFs (such as the Local Government Bodies, department of social welfare and public health engineering), were beyond the scope of this study. However, these directorates are doing a lot of public health works. Further studies need to be undertaken to document the challenges of these departments, particularly at the district level and below.

Our study has several strengths that contribute to the growing body of literature on public health functions. To our knowledge, it is the first to document the key stakeholders’ in-depth perspectives on what constitutes public health functions according to the sub-district and district-level health system in Bangladesh. In addition, the finding also might be necessary to health system researchers and policymakers on what practical challenges are there in performing those functions. Our research thus has the potential to contribute to the development of a set of essential public health functions in Bangladesh. Besides, this can help future policy-making initiatives on how to strengthen the public health workforce in Bangladesh. We recommend future research focusing on (i) understanding the modalities for delivering essential public health services at different tiers of the health system in Bangladesh and (ii) identifying the most effective policy response to integrate public health services across different departments of the ministry of health in Bangladesh.

In order to strengthen the health system of a country, appropriate emphasis needs to be placed on characterizing the public health functions and ensuring an appropriate workforce at all tiers of the health system. Especially, ensuring public health functions at the peripheral tier of a country’s health system not only helps to prevent disease outbreak but also helps deliver equitable and people-centric care. Our study findings suggest that although Bangladesh has an excellent country-wide network of healthcare delivery infrastructure, there is still a need to identify the essential public health functions for different health systems. Besides, to ensure these functions are being properly carried out, it is crucial to revisit the sanctioned posts and their job descriptions, especially for the physician cadre leading these services at the district and sub-district level health system. In this regard, careful policy attention is required to prioritize the public health functions that will ultimately help achieve the targets for health-related Sustainable Development Goals.

## Abbreviations

ANC: Antenatal Care
CDC: Centers for Disease Control and Prevention
CS: Civil Surgeon
DGDA: Directorate General of Drug Administration
DGFP: Directorate General of Family Planning
DGHS: Directorate General of Health Services
EPHF: Essential Public Health Functions
EPI: Extended Program on Immunization
FP: Family Planning
IEDCR: Institute of Epidemiology, Disease Control and Research
IRB: Institutional Review Board
KII: Key Informant Interview
LMICs: Low and Middle Income Countries
MODC: Medical Officer-Disease Control (MODC)
MOHFW: Ministry of Health and Family Welfare
MNCH: Maternal Neonatal and Child Health
NCD: Non Communicable Diseases
SBCC: Social Behavior Change Communication
SDGs: Sustainable Development Goals
SRQR: Standards for Reporting Qualitative Research
TB: Tuberculosis
UHFPO: Upazila Health and Family Planning Officer
UHC: Universal Health Coverage
WHO: World Health Organization
